# In silico analysis of mutations near S1/S2 cleavage site in SARS‐CoV‐2 spike protein reveals increased propensity of glycosylation in Omicron strain

**DOI:** 10.1002/jmv.27845

**Published:** 2022-06-07

**Authors:** Christopher A. Beaudoin, Arun P. Pandurangan, So Yeon Kim, Samir W. Hamaia, Christopher L.‐H. Huang, Tom L. Blundell, Sundeep Chaitanya Vedithi, Antony P. Jackson

**Affiliations:** ^1^ Department of Biochemistry, Sanger Building University of Cambridge Cambridge United Kingdom; ^2^ Department of Biochemistry, Hopkins Building University of Cambridge Cambridge United Kingdom; ^3^ Physiological Laboratory University of Cambridge Cambridge United Kingdom

**Keywords:** Delta variant, furin, O‐linked glycosylation, Omicron variant, SARS‐CoV‐2 spike protein, TMPRSS2

## Abstract

Cleavage of the severe respiratory syndrome coronavirus‐2 (SARS‐CoV‐2) spike protein has been demonstrated to contribute to viral‐cell fusion and syncytia formation. Studies have shown that variants of concern (VOC) and variants of interest (VOI) show differing membrane fusion capacity. Mutations near cleavage motifs, such as the S1/S2 and S2' sites, may alter interactions with host proteases and, thus, the potential for fusion. The biochemical basis for the differences in interactions with host proteases for the VOC/VOI spike proteins has not yet been explored. Using sequence and structure‐based bioinformatics, mutations near the VOC/VOI spike protein cleavage sites were inspected for their structural effects. All mutations found at the S1/S2 sites were predicted to increase affinity to the furin protease but not TMPRSS2. Mutations at the spike residue P681 in several strains, such P681R in the Delta strain, resulted in the disruption of a proline‐directed kinase phosphorylation motif at the S1/S2 site, which may lessen the impact of phosphorylation for these variants. However, the unique N679K mutation in the Omicron strain was found to increase the propensity for O‐linked glycosylation at the S1/S2 cleavage site, which may prevent recognition by proteases. Such glycosylation in the Omicron strain may hinder entry at the cell surface and, thus, decrease syncytia formation and induce cell entry through the endocytic pathway as has been shown in previous studies. Further experimental work is needed to confirm the effect of mutations and posttranslational modifications on SARS‐CoV‐2 spike protein cleavage sites.

## 1 INTRODUCTION

The severe respiratory syndrome coronavirus‐2 (SARS‐CoV‐2) has spread globally since the initial outbreak in Wuhan, China, in November 2019 and was declared the causative agent of the COVID‐19 pandemic in March 2020.^1^ Several variants of concern (VOCs) and variants of interest (VOIs) have since been discovered with mutations in the proteome that have resulted in differing virulence and transmissibility.^2^ Many of the recorded mutations, especially in that of the VOCs and VOIs, have been described to occur in the trimeric spike glycoprotein, which protrudes from the virion surface to engage with host cell receptors and induce viral cell entry.^3^ The differences in virulence related to mutations on the spike protein can be attributed to altered virus−host or intramolecular interactions: for example, antibody evasion, host cell receptor binding, and protein stability.^4^, ^5^ Thus, continuous monitoring and investigation of the effects of mutations in the spike protein may help provide a clearer understanding of changes in virulence throughout viral evolution.

The coronavirus spike protein has been described as being composed of two subunits, S1 and S2. The S1 subunit contains the N‐terminal and C‐terminal receptor‐binding domains, which bind to host cell receptors to initiate viral entry. After receptor‐binding, host proteases can cleave the spike protein at various motifs, which subsequently exposes an internal hydrophobic fusion peptide in the S2 subunit that binds and fuses the host and viral membranes.^6^ Two cleavage sites, S1/S2 and S2', have been shown to contribute to virus−host membrane fusion at the cell surface. Studies have suggested that cleavage at the S1/S2 site results in shedding of the S1 subunit and induces conformational rearrangement that, subsequently, allows the S2' site to become accessible to host proteases.^7^, ^8^

**Figure 1:**
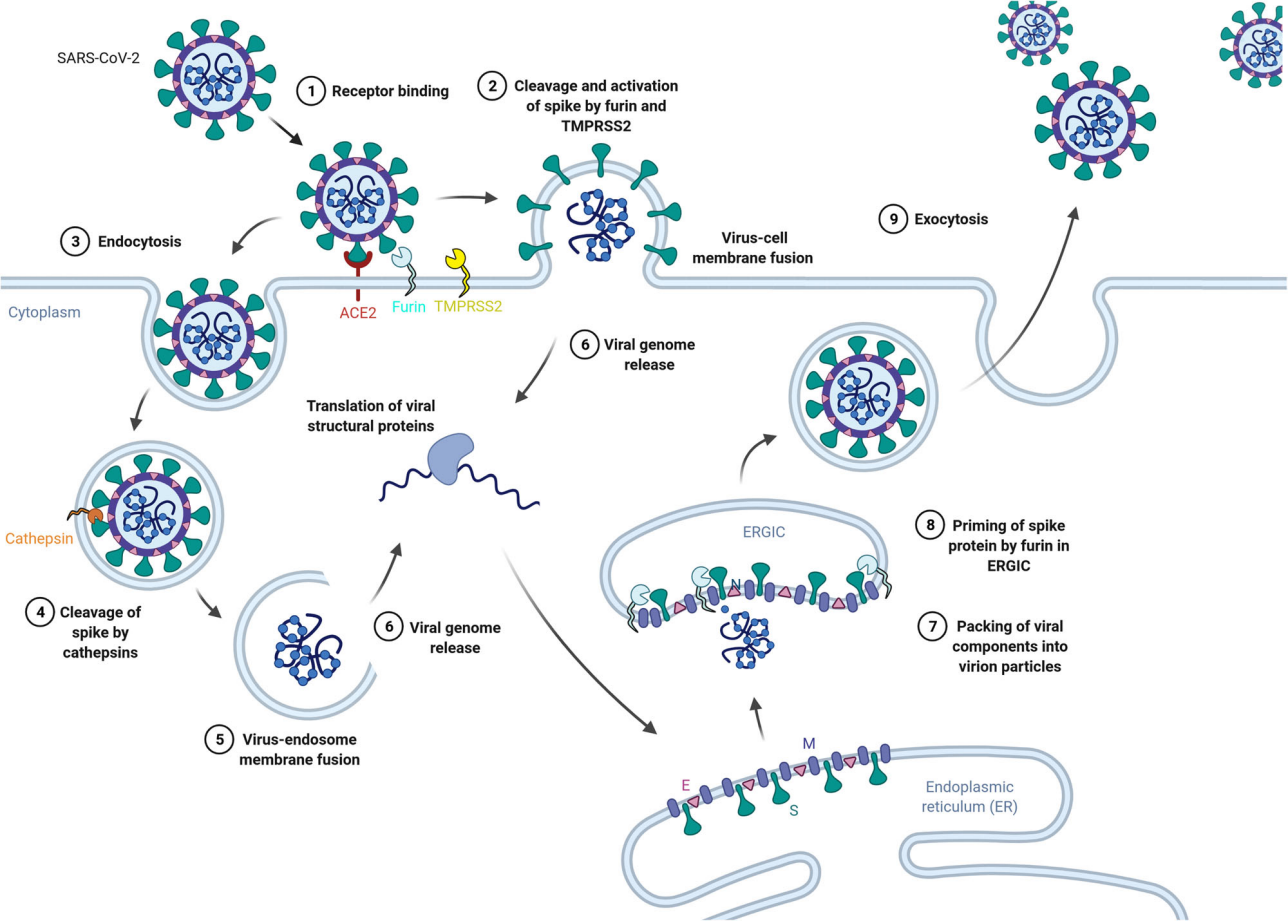
Pathways of viral cell entry for SARS‐CoV‐2 variants. Upon binding to the host cell receptor ACE2 (1), the SARS‐CoV‐2 spike protein can be cleaved at the S1/S2 site by furin, which induces conformational changes that expose the S2' site for cleavage by TMPRSS2 (2). Cleavage of the S1/S2 and S2' sites at the cell surface results in viral‐human cell fusion at the plasma membrane, (2) which releases the virion components, that is, single‐stranded RNA, into the cell cytoplasm directly (6). If cleavage of both sites does not occur at the cell surface after receptor binding, which characterizes the Omicron strain, then the virion may be endocytosed (3). Cathepsins within the endosome or lysosome can then be used to cleave the spike protein (4) to activate the spike protein and, thus, cause membrane fusion of the virus and endosome/lysosome (5), which releases the virion components into the cytoplasm (6). After replication of the genome and translation of viral proteins, the components move to the Endoplasmic reticulum (ER) and Golgi apparatus to be packed into virions (7). During the packing process, furin on the ER‐Golgi intermediate compartment membrane may cleave the spike protein at the S1/S2 site, which primes the virus for fusion during infection of the next host cell (8). The virus is then exocytosed (9). This figure was adapted from the “Coronavirus Replication Cycle” template, by BioRender (2022). Retrieved from https://app.biorender.com/biorender-templates. SARS‐CoV‐2, severe respiratory syndrome coronavirus‐2.

Unlike other known coronaviruses, the SARS‐CoV‐2 spike protein contains an insertion of an RRAR furin cleavage motif at the S1/S2 site, which has, indeed, been proven experimentally to confer cleavage by furin.^9^, ^10^ Since furin can be found intracellularly in the trans‐Golgi network, extracellularly on the plasma membrane, or secreted into the extracellular matrix, the spike protein can be cleaved and, thus, primed for fusion before or after viral particle assembly (Figure 1).^11^ Several proteases, such as TMPRSS2, MMP2/9, and neutrophil elastase, have been suggested to cleave the SARS‐CoV‐2 S1/S2 site as well.^12^, ^13^ Cleavage at the S2' site has been described to be cleaved by TMPRSS2 and related proteases. VOC/VOI mutations in and around the known cleavage sites on the spike protein have been shown to modulate viral−host membrane fusion capacity and kinetics.^14^ Additionally, studies have shown that deletions in the S1/S2 region hinder fusion at the cell surface and result, rather, in endocytosis of the virus—upon which cleavage by cathepsins can occur, albeit at a slower rate.^15^ Further analysis of the cleavage sites in the spike protein in relation to its variants may provide more information about infection dynamics.

Recently, the SARS‐CoV‐2 Omicron strain has been found to have little ability to undergo cell surface membrane fusion and, as a result, primarily invades the host cell through endocytosis.^16^, ^17^, ^18^, ^19^ Conversely, mutations throughout the spike protein in the other VOCs, such as Delta, and VOIs have been shown to increase fusion capacity.^14^ However, the biochemical basis for changes in interactions with proteases and their potential outcomes for membrane fusion based on these mutations has not yet been explored. Thus, herein, sequence‐ and structure‐based bioinformatics methodologies, such as post‐translational modification (PTM) prediction and protein−protein docking, were used to better understand the differences in structural features at the known cleavage sites in the VOCs/VOIs. Investigation of the interactions between spike proteins cleavage site and host proteases, such as furin and TMPRSS2, may shed light on the differential capacity to permit membrane fusion.

## 2 RESULTS AND DISCUSSION

### 2.1 Mutations near the S1/S2 and S2' cleavage sites in VOC/VOI spike sequences

**Figure 2:**
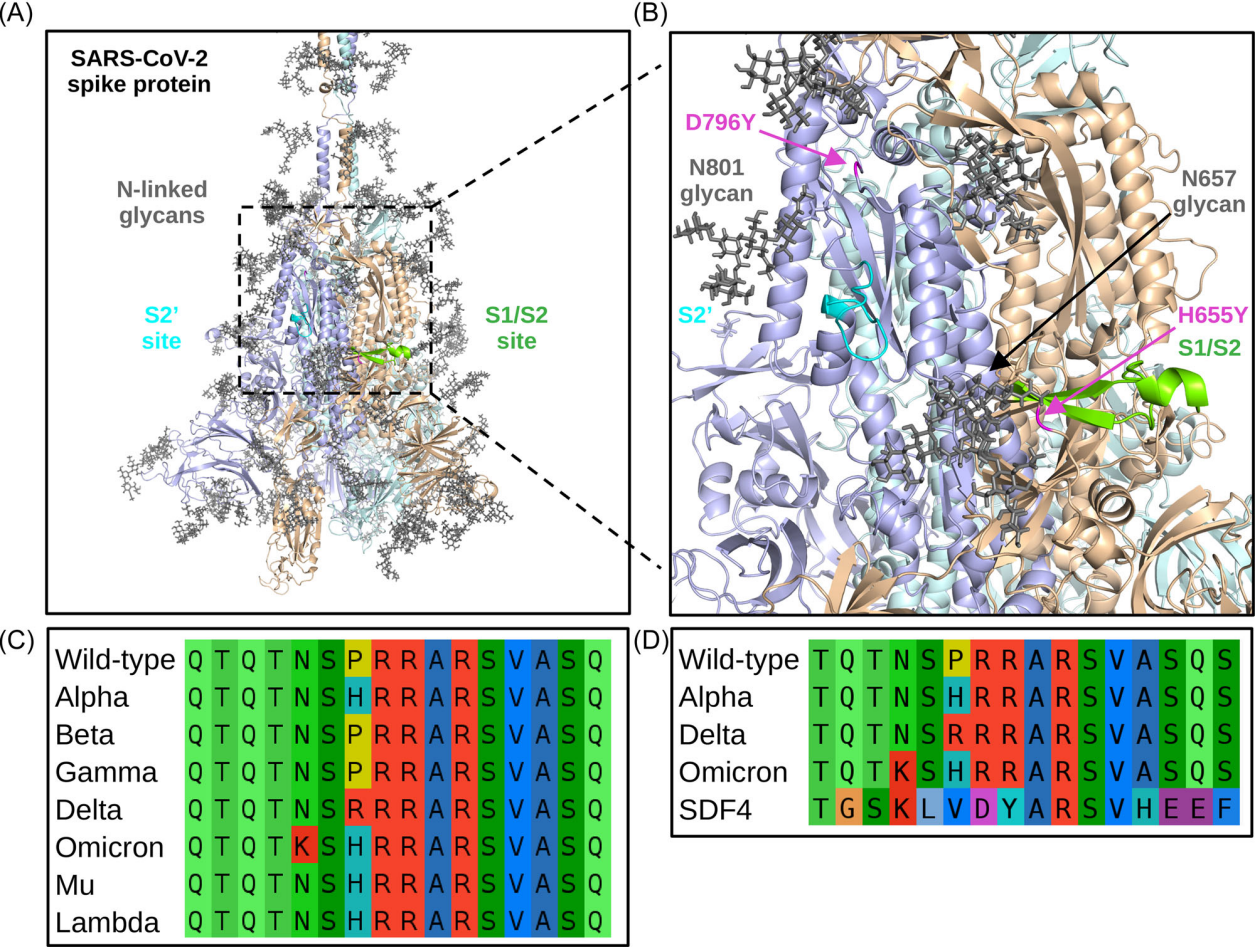
SARS‐CoV‐2 spike protein S1/S2 and S2' cleavage sites. The Wild‐type SARS‐CoV‐2 spike protein structure is shown colored by chain (tinted blue, orange, and green) with N‐linked glycans colored in the dark gray, the S1/S2 site colored in green, and the S2' site colored in cyan (A). A zoomed‐in image of the S1/S2 and S2' sites is depicted with noted Omicron mutations H655Y and D796Y shown in magenta (B). A sequence alignment of the S1/S2 site for the variants of concern and variants of interest is shown (C). A sequence alignment of the S1/S2 site of the Wild‐type, Alpha, Delta, and Omicron strains with the C‐terminal region of SDF4 is shown (D). SARS‐CoV‐2, severe respiratory syndrome coronavirus‐2.

The amino acid sequences of the spike protein from the VOCs (Alpha, Beta, Gamma, Delta, Omicron) and VOIs (Mu, Lambda) were selected to represent the most impactful mutations at S1/S2 and S2' sites (depicted in Figure 2A,B) and because these strains are more likely to have confirmatory experimental data. To better understand the position and context of the mutations, the variant sequences were aligned with the Wild‐type strain. As shown in Figure 2C, alignment of the Wild‐type and VOC/VOI sequences at the S1/S2 (Wild‐type spike residues: 675−690) and S2' (809−821) sites reveals three unique mutations around the S1/S2 site that have occurred, while no mutations near the S2' site were found in any VOCs or VOIs. Although mutations at two residues, H655Y and D796Y, somewhat near the S2' site were found on the Omicron strain, any potential impact on binding to proteases seems to be overshadowed by nearby N‐linked glycans at N657 and N801, respectively (Figure 2B). Thus, the S1/S2 motif was selected to better understand how mutations may affect interactions with host proteases.

Alignment of the S1/S2 site revealed that the P681R and N679K mutations occur in the Delta and Omicron strains, respectively, and the P681H mutation occurs in the Alpha, Omicron, Lambda, and Mu strains (Figure 2C). Among the aligned spike sequences, only the Omicron strain contains two mutations in the region concerned. Further investigation of the differences in the functional features based on the mutations may reveal their potential impacts on cleavage at the S1/S2 site.

### 2.2 PTM prediction

PTMs have been shown to be important modulators of protein−protein interactions.^20^ The decoration with 22 N‐linked glycans and at least one O‐linked glycan on each of three chains of the SARS‐CoV‐2 spike protein has been demonstrated to contribute to both receptor‐binding and antibody evasion.^21^ Additionally, the spike protein has been shown to be phosphorylated and palmitoylated.^22^, ^23^ Several bioinformatics tools have recently become available that accurately predict PTMs from protein sequences.^24^ Thus, to scan through the variant sequences to detect possible changes at the S1/S2 site as a result of the mutations, we used various sequence‐based PTM prediction methodologies.

#### 2.2.1 Glycosylation

No changes in N‐linked glycosylation were found to occur near the S1/S2 site. When comparing the O‐linked glycosylation predictions of the Wild‐type strain to experimental findings, the only validated site was T678, which scored highest (0.631) among the three predicted sites (Table 1).^25^ Thus, 0.631 was considered the prediction threshold. The other scores found above this threshold among the variant sequences were at T678 (0.672) and S686 (0.632) for the Gamma strain and at S683 (0.699) for the Omicron strain. The T678 on the Wild‐type and possibly on the Gamma strains is several amino acids from the known cleavage site but may have a partial blocking or stabilizing effect on host protease binding. The 0.699 score on the Omicron strain at the S683 residue reveals a high predicted propensity for O‐linked glycosylation at the S1/S2 cleavage site—occurring at the serine following the arginine used to cleave at this site. Such glycosylation may at least partially explain the experimentally‐observed decreased viral‐cell fusion and syncytia after infection with the Omicron strain. The P681R and P681H mutations were shown to decrease propensity for O‐linked glycosylation, which is confirmed by experimental work from Jun et al.^14^

**Table 1:** Sequence‐based PTM predictions

Strain	AA	Motif	NetOGlyc score	NetPhos score	NetPhos kinase	Musite score
Wild‐type	S673	GICASYQTQ	0.589			
	T678	YQTQTNSPR	0.631			
	S680	TQTNSPRRA		0.99	Unspecific	(P) 0.864
				0.586	Cdk5	
				0.55	p38MAPK	
	S686	RRARSVASQ	0.577	0.988	Unspecific	(P) 0.820
				0.62	PKA	
				0.541	PKG	
				0.525	PKC	
				0.514	RSK	
Alpha	S670	GICASYQTQ	0.507			
	S683	RRARSVASQ	0.500	0.934	Unspecific	(P) 0.806
				0.66	PKC	
				0.595	PKA	
				0.5	RSK	
Beta	S670	GICASYQTQ	0.500			
	T675	YQTQTNSPR	0.624			
	S677	TQTNSPRRA		0.99	Unspecific	(P) 0.864
				0.586	Cdk5	
				0.55	p38MAPK	
	S683	RRARSVASQ	0.587	0.988	Unspecific	(P) 0.809
				0.62	PKA	
				0.541	PKG	
				0.525	PKC	
				0.514	RSK	
Gamma	S673	GICASYQTQ	0.590			
	T678	YQTQTNSPR	0.672			
	S680	TQTNSPRRA		0.99	Unspecific	(P) 0.864
				0.586	Cdk5	
				0.55	p38MAPK	
	S686	RRARSVASQ	0.632	0.988	unsp	(P) 0.820
				0.62	PKA	
				0.541	PKG	
				0.525	PKC	
				0.514	RSK	
Delta	S671	GICASYQTQ	0.511			
	S684	RRARSVASQ	0.581	0.994	Unspecific	(P) 0.853
				0.783	PKB	
				0.738	PKC	
				0.577	RSK	
				0.531	PKA	
	S687	RSVASQSII		0.535	Cdk1	(P) 0.625
Omicron	S670	GICASYQTQ	0.609			
	K676	QTQTKSHRR				(M) 0.503
	S683	RRARSVASQ	0.699	0.934	Unspecific	(P) 0.803
				0.66	PKC	
				0.575	PKA	
				0.507	RSK	
Mu	S674	GICASYQTQ	0.523			
	S687	RRARSVASQ	0.516	0.66	PKC	(P) 0.806
				0.595	PKA	
				0.5	RSK	
Lambda	S666	GICASYQTQ	0.529			
	T671	YQTQTNSPR	0.580			
	S679	RRARSVASQ	0.509	0.988	Unspecific	(P) 0.820
				0.62	PKA	
				0.541	PKG	
				0.525	PKC	
				0.514	RSK	

Abbreviation: PTMs, posttranslational modifications.

To further examine the high score for the Omicron strain S683, the spike S1/S2 sequences were mapped to the sequences used in the O‐linked glycosylation detection tool (NetOGlyc) training data.^26^ Interestingly, only one similar sequence was found: the C‐terminal region of the 45 kDa calcium‐binding protein (SDF4). As shown in Figure 2D, an alignment of the spike sequences to SDF4 revealed (1) an identical ARSV motif, which permits O‐linked glycosylation on the serine residue on SDF4, to all SARS‐CoV‐2 sequences and (2) that the N679K mutation present on the Omicron strain increases sequence identity to SDF4. Examining the aligned region on the SDF4 AlphaFold2 3D model revealed that the motif is in an unstructured region of the protein, similar to the spike S1/S2 site.^27^ Thus, increased similarity of the Omicron strain S1/S2 site to a region on a human protein with validated O‐linked glycosylation may enhance the possibility of glycosylation. Of note, furin cleavage sites in human proteins have been found to be modulated by O‐linked glycosylation.^28^

Zhang et al.^29^ found that only one of several tested N‐acetylgalactosaminyltransferases (GALNT), GALNT1, efficiently catalyzes O‐linked glycosylation at the S1/S2 site on the SARS‐CoV‐2 spike protein. Checking publicly‐available proteomics and transcriptomics data on GALNT1 in healthy human tissues revealed that the gene is expressed in moderate amounts in cells from both the upper (tonsils and nasopharynx) and lower (bronchus and lung) respiratory tracts (Supporting Information: Table 1).

#### 2.2.2 Phosphorylation

Phosphorylation has been reported to occur at the SARS‐CoV‐2 spike protein S1/S2 site, which has also been shown to inhibit binding to proteases.^30^ Proline‐directed and basophilic protein kinases, which recognize SP and RXXS motifs, respectively, were found to recognize the SP and RARS motifs on the Wild‐type spike protein. Thus, comparing predicted phosphorylation sites between spike protein variants might help reveal the differences as a result of mutations.

As shown in Table 1, the Wild‐type S1/S2 site was found to be phosphorylated at the S680 and S686 residues, which is confirmed by the aforementioned experimental data. Although both residues score highly in their general capacity to be phosphorylated, the S680 is predicted to be phosphorylated by Cdk5 and p38MAPK and S686 by PKA, PKG, PKC, and RSK. These results correlate well with the experimental data that show that Cdk1 and PKA can phosphorylate the Wild‐type S1/S2 site, although Cdk5 as opposed to Cdk1 was preferred to phosphorylate the S680 residue in the predictions. Phosphorylation was predicted to occur at two sites (S680 and S686 on Wild‐type spike) in the Wild‐type, Beta, and Gamma strains, while only one site (S686 on Wild‐type spike) was found for the Alpha, Omicron, Mu, and Lambda spike proteins. The Delta strain was also predicted to have two phosphorylation sites (S686 and S689 on Wild‐type spike), but phosphorylation of the S687 (S689 on the Wild‐type spike) is unique in relation to the other strains. Since the mutations at P681 at the S1/S2 site result in substitution of the proline residue at the proline‐directed SP motif, several of the variants were predicted not to phosphorylate the upstream S680 as expected. Although the predicted kinases differ slightly between the variant spikes, PKA, and PKC were predicted to phosphorylate the serine corresponding to the Wild‐type S686 on all variant spike proteins. The S686 residue is directly at the cleavage site and, thus, may prevent interactions with proteases from occurring at this site.

Checking the expression levels of PKA, PKC, Cdk1, and Cdk5 revealed that PKA and Cdk5 are found at higher levels than PKC and Cdk1, respectively, throughout the respiratory tract, although all kinases were found to be expressed in the respiratory epithelium (Supporting Information: Table 1).

Interestingly, a previous study also found that the S816 residue of the S2' cleavage site can be phosphorylated in the Wild‐type spike protein.^23^ Thus, further investigation of the phosphoregulation at spike protein cleavage sites and the differences between variants may provide insights into viral fusion and virulence.

#### 2.2.3 Other PTMs

No acetylation, palmitoylation, or SUMOylation was detected at the S1/S2 sites for all spike sequences. Methylation was predicted to occur just above the MusiteDeep threshold at the lysine in the N679K mutation on the Omicron spike protein (Table 1). To our knowledge, no study has yet reported the potential for methylation at the S1/S2 site. Such methylation may affect the charge and sterics of this residue and, thus, binding to host proteases.

Altogether, the potential presence of PTMs, such as O‐linked glycosylation may prevent interactions with host proteases and, thus, cleavage from occurring at the S1/S2 site. Interestingly, a previous study has shown that abrogation of furin cleavage by O‐linked glycosylation can be inhibited by phosphorylation of the same residue, thus permitting cleavage.^31^ Therefore, further investigation of the interplay between phosphorylation, glycosylation, and other PTMs at the S1/S2 site may clarify their effects on protease recognition. Additional computational and experimental monitoring of PTMs at cleavage sites is needed to better understand their impact. Analysis of the structural impacts of the S1/S2 mutations in the context of their interactions with host proteases may further delineate differences in cleavage capacity between variants.

### 2.3 Structural implications of mutations and predicted PTMs at the S1/S2 cleavage site

Since the Alpha (P681H), Delta (P681R), and Omicron (P681H, N676K) sequences illustrate the variety of mutations at the S1/S2 site in all VOC/VOIs, they were selected for comparative structural analyses. Furin and TMPRSS2 were selected as host proteases for the protein−protein docking on account of their known involvement and available experimentally‐derived structures. As a result of the high flexibility of the S1/S2 site, no study utilizing cryo‐EM or X‐ray crystallography has been able to define a stable structure for this site. Thus, we used the full‐length spike model made by Woo et al.^21^ as a template for homology‐based modeling. Potential changes in affinity and interactions between the S1/S2 site and host proteases were investigated using protein−protein docking. The structural impacts of the predicted glycosylation and phosphorylation at the S1/S2 site were examined by modeling the respective structures and subsequently performing protein−protein docking to note differences in affinity and access to the cleavage site. Comparing biochemical interaction profiles between host proteases and the S1/S2 site of the spike protein variants may help further explain differences in cleavage capacity.

#### 2.3.1 Ensemble docking of spike protein S1/S2 site to host proteases

Ensemble docking has been found to be useful for understanding protein−protein interactions in which one or more of the protein interfaces is flexible or found in different conformations.^32^ Since the S1/S2 site is flexible, ensembles of conformations of the S1/S2 site of each spike protein were docked to the catalytic sites of the two proteases to better understand the overall binding capacity. Alongside the resulting docking score (ZDOCK score), predicted affinity (ΔG [kcal/mol]) and interface residues determined by the PRODIGY web server were used to compare the docked models. The changes in number of interacting residues with residue 681, with respect to the Wild‐type strain, was used to delineate the effect of mutations on interactions with proteases at the S1/S2 site. The ensemble dockings provide a broad overview of the potential binding modes and capacity between the spike variants and host proteases.

**Figure 3:**
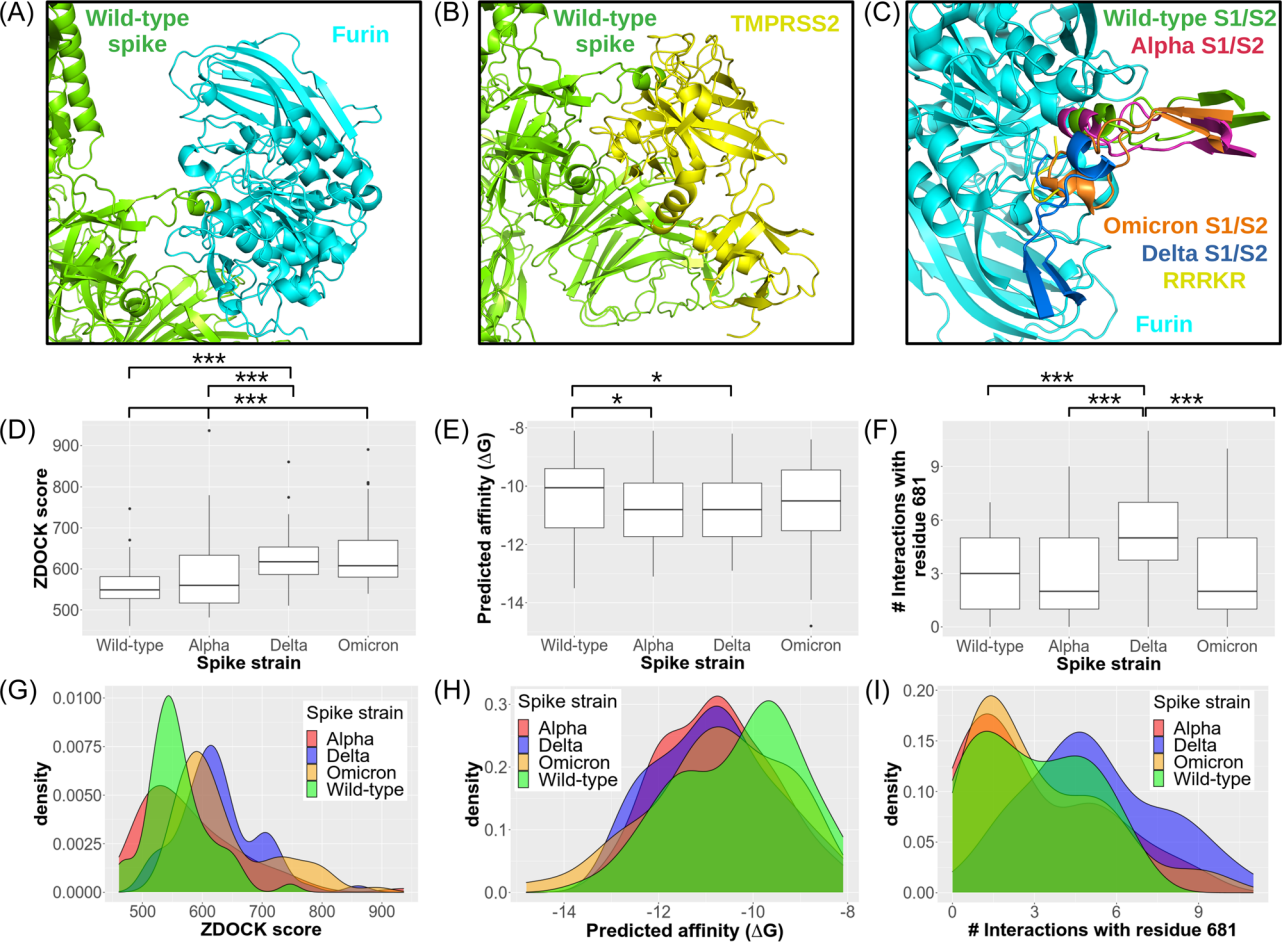
Structural effect of S1/S2 mutations on protease affinity. The top models of the docking of the Wild‐type spike protein S1/S2 (green) site to furin (cyan) (A) and TMPRSS2 (yellow) (B) is shown. Differences in orientation of the top models from the docking of the Wild‐type, Alpha (red), Delta (blue), and Omicron (orange) is shown (C). The RRRKR peptide from the furin PDB file is also shown (yellow) (C). The ZDOCK scores (D, G), predicted affinity (E, H), predicted interactions with residue 681 (F, I) calculated on models of the Wild‐type, Alpha, and Delta S1/S2 sites docked to furin are represented as box plots (D−F) and density plots (G−I). Statistical significance is noted with asterisks: * < 0.05, *** < 0.001.

The top‐ranked models of the Wild‐type S1/S2 site docked to furin and TMPRSS2 were predicted in similar orientations as have been previously demonstrated (Figure 3A,B).^33^ The ZDOCK scores were found to differ significantly between docking of furin to the S1/S2 sites of Delta and Wild‐type (*p* = 1.27 × 10^−8^), Delta and Alpha (*p *= 2.53 × 10^−3^), Omicron and Wild‐type (*p *= 1.62 × 10^−47^), and Omicron and Alpha (*p *= 5.98 × 10^−4^) (Figure 3D,G). A significant increase was found between the predicted affinity of furin with both Alpha and Delta cleavage sites when compared to the Wild‐type (respective *p* = 0.043, 0.033) but not between the Omicron and Wild‐type (*p *= 0.094) (Table 2; Figure 3E,H). In comparing the docked models of the template (nonconformer) S1/S2 sites to furin, the P681H mutation in the Alpha S1/S2 site rescued the Wild‐type docking orientation while the P681R mutation in the Delta S1/S2 site and the combined P681H and N676K mutations in the Omicron shifted the S1/S2 site towards acidic residues in the furin catalytic site (Figure 3C). Interestingly, the Delta and Omicron S1/S2 site orientations resemble that of the crystal structure of a cyclic RRRKR peptide bound to furin. In looking at the average number of interactions for the P681 mutations, the P681R mutation was found to significantly increase the number of interacting residues than the Wild‐type, Alpha, or Omicron S1/S2 sites (*p* = 6.89 × 10^−7^) (Figure 3F,I). These results reflect computational and experimental reports that state that the P681H and P681R mutations increase affinity to and cleavage capacity by furin.^34^, ^35^, ^36^, ^37^

**Table 2:** Docking scores between furin and phosphorylated (P) and non‐phosphorylated spike variant S1/S2 sites

Spike strain	ZDOCK score	Affinity (kcal/mol)	# Interactions with residue 681
Wild‐type	559.33 ± 52.18	−10.30 ± 1.28	2.95 ± 2.08
Wild‐type (P)	537.11 ± 43.97	‐	‐
Alpha	582.90 ± 87.20	−10.7 ± 1.16	3.00 ± 2.56
Alpha (P)	579.6 ± 99.24	‐	‐
Delta	626.80 ± 67.14	−10.80 ± 1.24	5.17 ± 2.52
Delta (P)	635.38 ± 64.64	‐	‐
Omicron	637.12 ± 81.03	−10.72 ± 1.44	3.17 ± 2.44
Omicron (P)	650.22 ± 64.12	‐	‐

No significant differences were found between the predicted scores and number of residue 681‐interacting residues for ensemble docking of the Wild‐type, Alpha, Delta, and Omicron S1/S2 sites to TMPRSS2 (Supporting Information: Table 2). Although the affinities did not significantly differ when comparing furin and TMPRSS2 dockings to one another, less poses were reported for docking of the all spike variant S1/S2 site conformers to TMPRSS2, which was interpreted as having less structural complementary and, thus, affinity to the S1/S2 cleavage sites.^38^ Further experimental work is required to assess the structural features of these mutations and their effects on cleavage at this site.

#### 2.3.2 Structural effects of glycosylation on host protease binding to spike S1/S2 site

**Figure 4:**
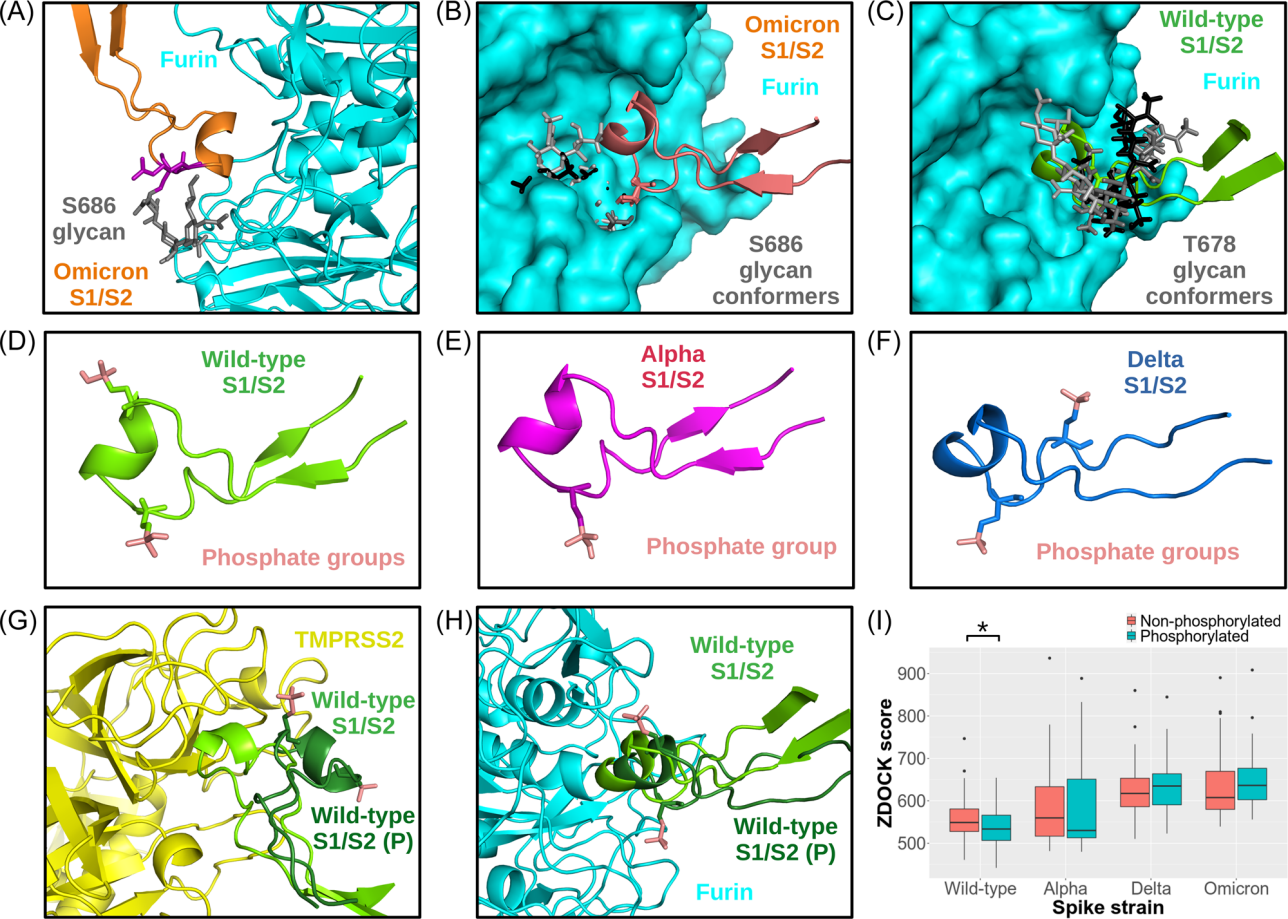
Structural effect of PTMs at S1/S2 site on protease recognition and affinity. The Omicron S1/S2 site modeled with the O‐linked glycan at S683 is shown docked to furin (A). The R and S residues involved in the cleavage (magenta) are shown facing away from the furin catalytic site. The glycated Omicron S1/S2 site (B) and Wild‐type S1/S2 site modeled with an O‐linked glycan at T678 (C) were aligned to the Wild‐type S1/S2 site docked to furin to show potential clashes with furin binding. The Wild‐type (D), Alpha (E), and Delta (F) S1/S2 sites are depicted with modeled phosphate groups. The models of the non‐phosphorylated and phosphorylated Wild‐type S1/S2 site is shown docked to TMPRSS2 (G) and furin (H). Since Alpha and Omicron both have only one predicted phosphorylation site, only Alpha is shown. A box plot comparing the ZDOCK scores of the non‐phosphorylated and phosphorylated Wild‐type, Alpha, and Delta S1/S2 sites to furin is shown (I). Statistical significance is noted with asterisks: * < 0.05. PTMs, posttranslational modifications.

In examining the effect of glycation on the S1/S2 site, both proteases were docked to the Omicron spike with an O‐glycan at S683 and the Wild‐type spike at S678. The Omicron spike glycan was found to completely block access to any interactions with the R685 or S686 residues (Figure 4A). As shown in Figure 4B, the glycan structure clashes directly with the proteases—irrespective of the glycan conformation—when aligned to the Wild‐type conformation. Thus, putative glycosylation at this residue on the Omicron strain may help explain the lowered capacity for fusion. Furthermore, such glycosylation may inhibit interactions with host receptors, such as neuropilin‐1, that bind directly to the S1/S2 site, as opposed to the spike receptor‐binding domains, to allow for viral cell entry.^39^, ^40^ The O‐glycan at T678 on the Wild‐type strain, however, was predicted to have minimal, if any, blocking effect—the non‐glycosylated docking orientations were rescued. As shown in Figure 4C, the Wild‐type O‐glycan is almost completely out of range of the predicted protease binding residues. Thus, glycosylation at this residue may have little effect on cleavage. Further experimental work is needed to confirm these findings.

#### 2.3.3 Structural effects of phosphorylation on host protease affinity to spike S1/S2 site

Phosphates were modeled at the predicted sites for the Wild‐type (S680, S686), Alpha (S683), Delta (S684, S687), and Omicron (S683) spike structures (Figure 4D–F) and redocked to the proteases. The modeled phosphates were found to completely prevent restoration of the template binding orientation or access to the R and S residues when docked to TMPRSS2 (Figure 4G). Docking to furin was able to reproduce the non‐phosphorylated docking modes (Figure 4H). Interestingly, the phosphorylated Wild‐type docking scores were slightly but significantly lower than non‐phosphorylated scores, while the Alpha, Delta, and Omicron docking scores were largely unaffected (Table 2; Figure 4I). These results suggest that phosphorylation of the Alpha, Delta, and Omicron S1/S2 sites may have a smaller effect on protease‐spike affinity than of the Wild‐type. However, the phosphorylation at S686, which is part of the cleavage site, may disrupt biochemical interactions necessary for cleavage that may not be completely captured by rigid‐body docking.^41^ Thus, phosphorylation at S686 may prevent cleavage at the S1/S2 site for all spike protein variants. Nevertheless, these results support the findings that phosphates at this site inhibit cleavage and suggest that mutations that lower the propensity of phosphorylation from occurring at this motif may allow for more efficient interactions with proteases.^30^ Further experimental work is needed to confirm these findings and validate the structural effect of phosphorylation on cleavage at the S1/S2 site.

## 3 CONCLUSIONS

Cleavage of the SARS‐CoV‐2 spike protein is an important virulence factor for viral cell entry and syncytia formation. While mutations in the Delta and Alpha strains have been shown to increase fusion capacity, the Omicron strain has been particularly shown to have lessened capacity for cleavage that allows for cell surface virus−host membrane fusion.^17^, ^35^ Rather, the Omicron strain has been suggested to enter the host cell through endocytosis—utilizing cathepsins to cleave the spike protein for later membrane fusion. To date, the biochemical mechanisms for these differences are largely unknown. Thus, sequence and structure‐based bioinformatics tools were utilized to better understand the effect of mutations on interactions with host proteases.

All mutations were found to potentially increase affinity between the S1/S2 site and furin, whereas the mutations were not found to increase affinity to TMPRSS2 at the S1/S2 site. The P681R mutation in the Delta strain, in particular, was predicted to significantly contribute to the increased interactions with furin. The N676K mutation in the Omicron spike protein may increase the propensity for O‐linked glycosylation at the S1/S2 site. Although the P681H and N676K mutations in the Omicron strain were predicted to increase affinity to furin—indicating that more efficient cleavage may occur in comparison to the Wild‐type—structural analyses suggest that such glycosylation may abrogate recognition by host proteases at the S1/S2 site. Since cleavage of the spike S1/S2 site has been suggested to be necessary for subsequent exposure and cleavage of the S2' site at the cell surface, the predicted glycosylation at S683 in the Omicron strain may explain the exhibited decreased viral‐host plasma membrane fusion and syncytia formation. However, potential glycosylation at the Omicron spike S683 may depend on the expression of appropriate GALNTs; thus, the effect of glycosylation on cleavage capacity could change depending on the infected tissues (e.g., cells in upper vs. lower airways). All mutations at the P681 residue were found to remove phosphorylation site at the S1/S2 site, which may prevent phosphorylation‐based inhibition of binding to proteases and, thus, increase fusion capacity for these variants. Experimental work is needed to confirm these results.

Mutations in regions outside of the S1/S2 and S2' sites, such as those near the fusion peptide, may also have impacts on fusion capacity and kinetics. Additionally, mutations near other protease sites, such as those found for cathepsins, may reveal differences in cell entry.^42^ Thus, further work is needed to characterize the structural differences in cleavage sites between spike protein variants for their effects on membrane fusion.

## 4 METHODS

### 4.1 Variant spike sequence information and comparison

Amino acid sequences of the Wild‐type (NCBI Accession: NC_045512), Alpha (MZ344997), Beta (MW598419), Gamma (MZ169911), Delta (MZ359841), Omicron (OL672836), Mu (OK005482), and Lambda (MW850639) strain spike proteins were obtained from the NCBI Viral Genomes Resource.^43^ Sequence alignments were performed with ClustalOmega.^44^ Alignments were visualized with AliView.^45^

### 4.2 PTMs prediction

Although several glycosylation prediction tools were tested, such as SPRINT‐Gly and GPP, the known experimental results are shown to be most aligned with NetNGlyc 1.0 and NetOGlyc 4.0 for predicting N‐ and O‐linked glycosylation, respectively, and, thus, they were used in the analysis.^26^, ^46^ NetPhos 3.1 was used to predict phosphorylation.^47^ The full list of potential PTMs from MusiteDeep were screened for each variant at the S1/S2 site with a threshold value of 0.5.^48^ Residues that were predicted to be phosphorylated by both NetPhos 3.1 and MusiteDeep were considered high‐confidence predictions, and sites found by only one of two tools were discarded. NetPhos 3.1 sites that corresponded to kinases found strictly in the nucleus, such as DNA‐PK, were excluded.

### 4.3 Expression data accession

Proteomics, RNA‐seq, and microarray data for tissue‐specific expression data from healthy human cells were obtained from the Human Protein Atlas and EMBL‐EBI Expression Atlas.^49^, ^50^ Only results from tissues in organs from the respiratory tract (bronchus, diaphragm, lung, nasopharynx, tonsil, and trachea) were selected.

### 4.4 Variant spike structure modeling

The Alpha, Delta, and Omicron spike homology models were generated using MODELLER and were subsequently refined using a molecular dynamics with simulated annealing protocol.^51^ The full‐length model made by Woo et al. was used as a template for the modeling. FoldX–RepairPDB was run on the spike models three times successively to repair torsion angles and reduce total energy.^52^ The O‐linked glycan template from Woo et al. and phosphate groups were modeled on the spike protein structures using the CHARMM‐GUI Glycan Modeler.^21^, ^53^ Structures were visualized using PyMol.

### 4.5 Ensemble protein−protein docking

Since the spike protein S1/S2 site is flexible and may be found at different conformational states before protease binding, we generated multiple conformations of the S1/S2 site for the Wild‐type, Alpha, Delta, and Omicron strains and docked them to furin (PDB: 6hzd) and TMPRSS2 (7meq). To generate representative conformers, the S1/S2 site of each spike protein was extracted (Wild‐type spike residues: C671‐Y695) from the structures and were submitted to the CABSFLEX 2.0 web sever, which utilizes molecular dynamics to measure flexibility and output structures at different conformations.^54^ Spike residues found only in the S1/S2 site were used for docking to proteases to determine predicted differences in affinity based on the mutations at this site alone. The conformers were aligned to the template using TM‐align and the five with the highest TM‐score and most accessible cleavage site were selected—totaling six conformers, including the template, for each spike variant.^55^ The S1/S2 conformers were subjected to protein−protein docking using ZDOCK, and residues surrounding the cleavage site (Wild‐type residues R682‐S689) were selected for interaction while others were selected for blocking (Wild‐type residues C671‐S680, Q690‐Y695).^56^ The top 10 poses for each conformer docking were selected to better understand the overall binding capacity of the spike S1/S2 sites to the proteases. The ZDOCK score and predicted affinity (ΔG [kcal/mol]) and interactions by the PRODGY web server were used to compare docked models.^57^ The Wild‐type and Omicron with the O‐linked glycans and phosphorylated Wild‐type, Alpha, and Delta S1/S2 site models were docked to furin and TMPRSS2 using ZDOCK to better understand the effect of PTMs on protease affinity and accessibility to the spike S1/S2 cleavage site. The PRODIGY web server was unable to accept phosphorylated proteins, thus only the ZDOCK score was used to compare docking of proteases with phosphorylated spike S1/S2 models.

### 4.6 Data analysis and availability

Statistical analyses and plots were generated using R version 3.6.3. Mean, standard deviation, and Student's *t*‐tests were used to calculate statistical differences between groups (*p *< 0.05 was considered significant; * < 0.05, ** < 0.01, *** < 0.001). All data and structural models are available at the tlb‐lab github (https://github.com/tlb-lab).

## AUTHOR CONTRIBUTIONS

Christopher A. Beaudoin, Arun P. Pandurangan, and So Yeon Kim performed sequence‐ and structure‐based analyses, summarized data, and wrote the paper. Samir W. Hamaia, Christopher L.‐H. Huang, Tom L. Blundell, Sundeep Chaitanya Vedithi, and Antony P. Jackson advised on methodologies, interpreted data, and wrote the paper.

## CONFLICT OF INTEREST

The authors declare no conflict of interest.

## Supporting information

Supporting information.

## Data Availability

The data that support the findings of this study are openly available in TLB‐lab github at https://github.com/tlb-lab/Coronavirus_spike_variant_cleavage.
